# From Nucleus to Organs: Insights of Aryl Hydrocarbon Receptor Molecular Mechanisms

**DOI:** 10.3390/ijms232314919

**Published:** 2022-11-29

**Authors:** Claudia M. Rejano-Gordillo, Beatriz Marín-Díaz, Ana Ordiales-Talavero, Jaime M. Merino, Francisco J. González-Rico, Pedro M. Fernández-Salguero

**Affiliations:** 1 Departamento de Bioquímica y Biología Molecular y Genética, Facultad de Ciencias, Universidad de Extremadura, 06006 Badajoz, Spain; 2 Instituto Universitario de Investigación Biosanitaria de Extremadura (INUBE) Campus de Badajoz, Avenida de la Investigación s/n, 06071 Badajoz, Spain

**Keywords:** aryl hydrocarbon receptor, epigenetics, signaling pathways, organ homeostasis

## Abstract

The aryl hydrocarbon receptor (AHR) is a markedly established regulator of a plethora of cellular and molecular processes. Its initial role in the detoxification of xenobiotic compounds has been partially overshadowed by its involvement in homeostatic and organ physiology processes. In fact, the discovery of its ability to bind specific target regulatory sequences has allowed for the understanding of how AHR modulates such processes. Thereby, AHR presents functions in transcriptional regulation, chromatin architecture modifications and participation in different key signaling pathways. Interestingly, such fields of influence end up affecting organ and tissue homeostasis, including regenerative response both to endogenous and exogenous stimuli. Therefore, from classical spheres such as canonical transcriptional regulation in embryonic development, cell migration, differentiation or tumor progression to modern approaches in epigenetics, senescence, immune system or microbiome, this review covers all aspects derived from the balance between regulation/deregulation of AHR and its physio-pathological consequences.

## 1. Introduction

**Figure 1:**
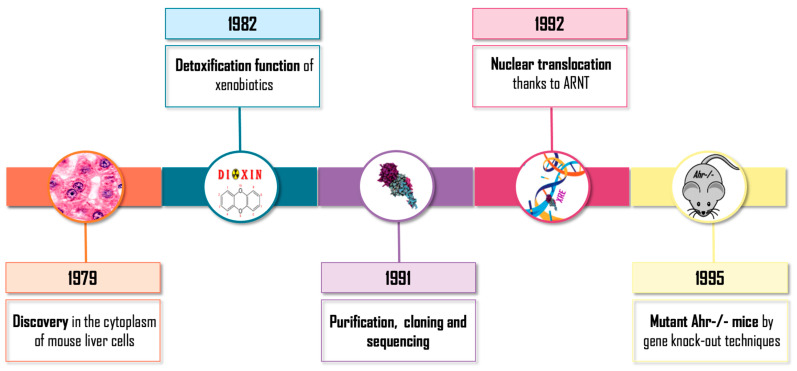
Early timeline of AHR research.

Intensive and uninterrupted research on aryl hydrocarbon receptor (AHR) emerged in the 1970–1980s (Figure 1). Initially, AHR was discovered in the cytoplasm of mouse liver cells. It was defined as a protein involved in the detoxification of xenobiotics due to its involvement in the induction of detoxifying enzymes such as cytochrome P450 CYP1A1 [1,2]. During the following years, the basic molecular mechanism of action of the receptor was traced, the translocation to the nucleus of the receptor was described as being dependent on its binding to exogenous ligands (generally polycyclic aromatic hydrocarbons) [3] and its relationship with the protein ARNT (aryl hydrocarbon receptor nuclear translocator) was uncovered [4]. Afterwards, its purification, cloning and sequencing was carried out, which allowed it to be considered a member of the superfamily bHLH/PAS of transcription factors with sequences of the basic-Helix-Loop-Helix (bHLH) type of class VII [5,6], which also includes the PAS domain transcription regulators: Per (Period), ARNT and Sim (Single Minded). Finally, the production of mutant mice by gene knock-out techniques was achieved, confirming the toxicological role of the receptor: AHR-/- mice were resistant to the toxic and carcinogenic effects of xenobiotics such as dioxin TCDD (2,3,7,8-tetrachloro-dibenzo-p-dioxin) and Benzo[a]pyrene (BP) [7,8,9]. Those initial investigations based on AHR knock-out murine models revealed an unexpected molecular and physiological role for the receptor, with a strong implication in cell cycle, proliferation, apoptosis and carcinogenesis [10].

Most of the studies on the transcriptional activity of AHR have focused on the activation mediated by the binding of exogenous ligands. Once translocated to the nucleus, it has the ability to bind to DNA [11,12,13] in consensus sequences called XREs or DREs (xenobiotic/dioxin response elements) in the promoter regions of its target genes, which contributes to increase or repress their transcription [14,15]. Consequently, the regulation of such transcriptional activity in the absence of xenobiotics is one of the most thrilling aspects of the functionality of this receptor.

## 2. Epigenetics and Chromatin: AHR-Driven Barriers

Non-coding regions represent about 98% of the human genome. The biological and evolutive argument behind this vast percentage contains regulatory elements that control gene expression such as promoters, enhancers, insulators, boundaries and post-transcriptional control elements. Together, all these regulatory elements allow the non-coding region of the genome to maintain the proper balance of gene expression that allows the cell an adaptive response to environmental and homeostatic stimuli [16]. In the last decade, this type of special regulation, far from the classic transcriptional regulation, has experienced a growing interest, with AHR presenting a pivotal role in the interaction of retrotransposons, chromatin spatial organization and epigenetic modifications [17].

### 2.1. Retrotransposons

The human genome comprises approximately 40% mobile elements that includes retrotransposons (retrotransposable elements, RTEs), DNA transposons and elements of retroviral origin, which potentially play a role in this whole mechanism. The transposons include short interspersed nuclear elements (SINE), long interspersed nuclear elements (LINE) and long terminal repeat (LTR) [18,19]. Several years have passed since these elements were molecularly and genetically described [20,21] until their functional implications in physiology and development were partially addressed [22]. Bioinformatic analysis confirmed that a high percentage of the binding sites for transcription factors established in the mammalian genomes resided in repetitive elements and, additionally, they were grouped in small regions forming similar structures to the cis elements that regulate genes, sharing a common expression pattern [23], allowing for a potential fast response to abrupt environmental changes. The group of SINEs includes the B1 and B2 elements from the mouse genome, named for their homology with double-stranded regions of the dsRNA-B nuclear pre-mRNAs [24], as well as their equivalent human Alu elements, which are named for the presence of a cleavage site for the AluI restriction enzyme [25,26]. Interestingly, both B1 and Alu elements present potential AHR binding sites [27,28]. In the mouse genome, the repetitive element B1 × 35S presents a functional XRE site for AHR binding within 35 bp from an E-box functional site for Slug/SNAI2 binding, resulting in the repression of several X35S-containing cis-reporter genes [27]. In fact, the transcription of this B1 × 35S element is dependent on an RNA polymerase switch triggered by AHR binding to the XRE site present on the retrotransposon itself, which includes the release of RNA Pol III and the recruitment of RNA Pol II [29]. On the other hand, in the human genome, bioinformatic analysis confirmed the presence of three B1 × 35S counterparts, with the Alu elements X45S, X36S and X14S having the XRE and SLUG/SNAI2 sequences separated by 45, 36 and 14 bp, respectively [28,30]. In these works, it has been described that, using an in vitro human differentiation model, AHR was able to bind to the Alu elements flanking stemness-relevant genes such as NANOG and OCT4, whose promoters harbor AHR and RNA Pol III binding sites. Furthermore, repression of such genes would likely imply processing of Alu-derived transcripts through the miRNA machinery involving a microprocessor (DGCR8 and DROSHA) and RISC (RNA induced silencing complex) [28], showing a regulation of carcinoma cell differentiation via AHR-regulated Alu retrotransposons. Together, these results suggest the existence of an AHR-driven control of gene expression through modulation of retrotransposons activity.

### 2.2. Chromatin Structural Organization

Chromatin fold/unfold balance provides a mechanism of control of gene expression through changes in the spatial organization of the chromosomes. This creates the possibility for long range interaction between distant regions, allowing the formation of topologically associated domains (TADs) for such purpose [31,32,33,34,35]. In fact, the position that regulatory elements occupy throughout the genome (promoters, repressor elements, enhancers and insulators, among others) is key to such regulation. Regarding this, it has been described that AHR is able to form a chromatin loop that encases the NANOG locus through the binding to its Alu flanking retrotransposons, controlling its ratio of expression/repression [30]. Interestingly, subsequent proteomic analysis revealed a complex network of proteins recruited by this Alu–AHR axis, which are implied in the conformation of such a loop. This group includes the chromatin assembly factor 1 subunit B (CHAF1B) and the protein arginine methyltransferase 1 (PRMT1). Additionally, the activation of AHR during early embryo differentiation alters such process through nucleosome remodeling and deacetylation complex (NuRD), controlling the pluripotency program during development [36]. Consequently, these interactions confirmed the connection between AHR-driven chromatin conformational changes and other epigenetic marks, such as histone methylation [30]. This interaction was hinted in previous works with the downregulation of AHR by its promoter hypermethylation in acute lymphoblastic leukemia. Suchlike molecular mechanism is driven through the unbinding of transcription factor Sp1 from AHR promoter, suggesting an epigenetic mechanism for the control of AHR expression in human tumor cells [37]. Moreover, the histone methylation pattern from me3H3K4, me3H3K9 and me3H3K27 marks was altered in the absence of AHR both in the human and mouse genome [29,30], reinforcing the importance of AHR in this regulation. Additionally, regarding the position of regulatory elements through the genome, the mouse retrotransposon B1 × 35S presented insulator activity both in vivo and in vitro, being able to repress gene expression depending on its physical position between the enhancer and the promoter [29]. Interestingly, the mutation of the AHR and Slug/Snai2 binding sites translated in a loss of insulator activity [27]. This retrotransposon enhancer blocking activity has emerged as a novel regulation of gene expression in recent years [38,39], being also very similar to the genomic control exerted by CCCTC-binding factor (CTCF) due to its insulator capacities [40,41]. In fact, CTCF is able to promote the formation of chromatin loops that modulate the accessibility of RNA polymerases and related proteins to gene regulatory regions, contributing to this chromatin barrier regulatory network [42,43,44]. Moreover, binding of CTCF also provides a physical anchor point for nucleosome positioning and chromatin remodeling [45]. The cooperation between CTCF and AHR initially addressed in mouse retrotransposon regulation [29] was later also confirmed in human Alu elements [30]. Thereby, the retrotransposons X45S and X14S showed a notably enhanced blocking activity, being AHR and CTCF co-recruited for the retrotransposon-propelled cell differentiation through NANOG locus silencing.

The novel findings reported in the molecular mechanisms underlying chromatin structural reorganization have been considered a milestone due to the non-canonical nature of its regulation. The implication of AHR in such processes remains a promising path to travel in the future of epigenetics.

## 3. AHR Signaling

Beyond the role of AHR in transcriptional regulation, its implication in multiple signaling pathways has built a field of study regarding its participation in the control and maintenance of different physiological and pathological processes [46]. The variety of responses resulting from AHR activation may be due to the different interactions of the receptor with other proteins or transcriptional cofactors [47]. Therefore, numerous proteins affecting AHR activity and vice versa have been described over the years, constituting a solid trend in cell signaling.

### 3.1. The Wnt/β-Catenin Pathway

The importance of this pathway resides in its participation in the correct development of most organs [48,49,50]. It has been described that Wnt-mediated signaling needs to be in balance in order to obtain a proper development program, as both downregulation and over-signaling of Wnt can lead to several defects [51,52,53], including lung, breast and skin cancer [54].

Despite AHR and Wnt signaling have been long studied independently, numerous evidence of crosstalk between the two pathways have emerged in recent years [55]. Interestingly, it has been described that AHR and Wnt/β-catenin cooperate in the induction of AHR transcriptional targets, such as Cyp1a1 and Cyp1b1, with the persistent AHR activation triggering reduced levels of active β-catenin, affecting the phenotype of hepatic progenitor cells and leading them towards other more differentiated types [56]. Furthermore, AHR agonists such as indole-3-carbinol (I3C), 3,3′-diindolylmethane (DIM) and indirubin-3′-monoxime were able to downregulate CTNNB1(β-catenin) expression [57,58,59].

Subsequently, AHR was identified as an inhibitor of the canonical Wnt signaling pathway in mouse intestine, being able to suppress intestinal carcinogenesis by degradation of β-catenin [60], with activated AHR expression also downregulating CTNNB1 expression in human colon cancer cell lines [55]. In the human breast cancer cell line, constitutive expression of AHR by introduction of a mutation was able to negatively regulate CTNNB1 [55]. Furthermore, in mouse liver, elevated levels of β-catenin were detected in AHR -/- preweaning mice [61]. This alteration could be possible due to AHR being part of the repressive complex that binds to CTNNB1, promoting its ubiquitination and subsequent degradation [55,60], since both proteins co-immunoprecipitated under basal conditions in adult liver [61]. AHR ligands also decrease ABC levels and generate further alterations in the Wnt pathway, such as Dvl dephosphorylation [56]. On the other hand, TCDD-induced reduction of the canonical Wnt pathway has been associated with a decrease in R-Spondin2 (Rspo2) and R-Spondin3 (Rspo3) activators [62]. However, in zebrafish, AHR activation by TCDD was shown to upregulate canonical Wnt signaling by overexpression of R-Spondin1 (Rspo1) [63]. Such Rspo1 signaling is mediated by the co-receptor LRP6 (low-density lipoprotein receptor-related protein 6), which is required for TCDD to upregulate the Wnt pathway and inhibit regeneration in zebrafish. This is consistent with data indicating that the role of AHR is cell type, tissue, organ and animal model dependent [64,65,66].

### 3.2. The PI3K/AKT Pathway

The phosphatidylinositol 3-kinase (PI3K)/protein kinase B (AKT) signaling pathway is one of the key regulators of cell proliferation, cell cycle and apoptosis [67]. Moreover, its over activation and de-regulation is a common feature in human malignancies, making it an ideal candidate for drug-based cancer therapies [68].

The importance of AHR in such processes is notorious, presenting a relevant series of interactions with the pathway. Hence, AHR has been found to reduce apoptosis in a mouse hepatoma cell line due to increased AKT activation [69]. However, a recent in vivo study evidenced that there was more AKT activity in AHR -/- mouse liver, thus p-AKT target GSK3β is more efficiently phosphorylated in the liver of such knockout animals [61]. In fact, GSK3β constitutes a link between the Wnt-β-Catenin and PI3K/AKT pathways, being able to inhibit itself in the absence of AHR and ensuring the maintenance of cellular homeostasis. Furthermore, PI3K also was found to have a connection with the Ras pathway via regulation of the activation of mitogen-activated kinases (MAPKs), presenting a sustained PI3K-dependent ERK1/2 activation in AHR -/- mice liver [61,70].

AHR has also been reported to control AKT phosphorylation under basal conditions without the necessity to respond to growth factors, cigarette smoke extract (CSE) or AHR ligands. Due to the finding that in the absence of AHR there is differential phosphorylation of several proteins such as fibrillin and fibronectin, it is speculated that the increased phosphorylation of AKT in AHR-/-MLF (mouse lung fibroblast) could be associated with extracellular matrix (ECM) deregulation [71]. This apparent discordance between the results of different studies suggests that AHR regulation of basal AKT activity may be cell-type specific and/or reflect differences between primary and cancer cells, a well-known feature of AHR [17].

### 3.3. Interaction with TGF-β Signaling

Transforming growth factors β (TGF-β) are cytokines that have an important role in proliferation, development, homeostasis and tumorigenesis [72,73]. It is known that TGF-β can be activated by mechanisms involving proteolytic cleavage of latent TGF-β binding protein (LTBP-1) and the release of thrombospondin-1 (TSP-1) [72,74]. Once activated, it binds to its membrane receptors, initiating the signaling pathway [75]. Due to the processes that present TGF-β participation, its crosstalk with AHR has been widely studied. Regarding this, in a mouse fibrosis liver model, AHR-/- mice showed higher levels of LTBP-1 protein, co-localizing with TGF-β1 and collagen accumulation. This LTBP-1 could be responsible for TGF-β activation through alterations of the proteases PA/plasmin, elastase and TSP-1 (Thrombospondin 1), all of them affected by the presence or absence of AHR [75]. In fact, in mouse liver and mouse embryonic fibroblasts (MEFs) from AHR-/- mice, a higher level of total and active TGF-β versus the AHR+/+ ones was reported, leading to a reduced proliferation rate and increased apoptosis [14,75,76]. Furthermore, the AHR knockout mice also presented a better skin wound healing process than the wild type ones, which could be a consequence of the higher cell migration pattern triggered by the over-activation of TGF-β pathway [77,78].

Interestingly, the interaction between AHR and TGF-β seems to be tissue-dependent. In fact, while AHR is able to repress TGF-β signaling in brain tumors [79], TGF-β is also needed for the maintenance of proper AHR expression levels in lymphocytes [80]. Moreover, the tandem TGF-β/Smad can dissociate the AHR/ARNT complex through the inhibition of CYP1A1-mediated metabolic activation of polycyclic aromatic hydrocarbons (PAHs), resulting in cell protection against carcinogenesis [81].

### 3.4. NF-κβ and p65

The modulation exerted by AHR in the inflammatory response via the nuclear factor kappa enhancer of activated B-cell light chains (NF-κβ) has been widely studied. The acute inflammatory response in macrophages is mediated by the recognition of microbial products by toll-like receptors (TLRs), and its activity is controlled by NF-κβ and RelA/p65 [82]. It has been shown that the RelB subunit of NF-κβ is physically associated with AHR in U937 macrophage cell line, whereas in TCDD-treated Hepa1c1c7 cells it was seen that the physical association involved AHR and RelA/p65 [83,84]. In fact, it has been revealed the ability of AHR to suppress NF-κβ/p65 signaling pathway in intestinal epithelium [85]. Interestingly, a similar pattern was found in a model of inflammatory response in lung, where AHR presented a negative regulatory capacity of such response through direct modulation of NF-κβ signaling [86]. Additionally, this association of AHR with RelA has been found to activate NF-κβ activity in order to upregulate interleukin-6 (IL-6) expression [87]. Consequently, there is thus a crosstalk between the NF-κβ and AHR pathways, in such a way AHR activation favors RelA/p65 protein degradation by ubiquitin–proteasome system (UPS) and lysosomes, resulting in decreased levels of proinflammatory cytokines in mouse macrophages [88]. In fact, such crosstalk between AHR and NF-κβ pathways could contribute to a variety of AHR responses during the different types and stages of chronic kidney disease (CKD) [89].

Therefore, the interaction between AHR, RelA, RelB and NF-κβ signaling pathways continues to be an interesting field of study since it was only initially unveiled [90].

### 3.5. Other Protein Interactions

Additionally, there are multiple proteins which has been described to affect AHR activity and vice versa. Their main interactions are highlighted in the Table 1.

## 4. AHR Physiological Functions

The initial approach on AHR research has been developed from a toxicological point of view due to the abusive use of xenobiotic compounds and their detrimental effect on human health in the last decades. However, numerous experimental evidences, mainly derived from the study of AHR knock-out mice, have revealed its physiological role, since its absence causes hepatic, reproductive, cardiac and immunological alterations [103]. In addition, several facts support the functional relevance of AHR in cell physiology: (i) its presence on the evolutionary scale long before that of polycyclic aromatic compounds in the atmosphere, (ii) its constitutive expression in most cell types, and (iii) its degree of conservation among various groups of both aquatic and terrestrial vertebrates [104].

**Figure 2:**
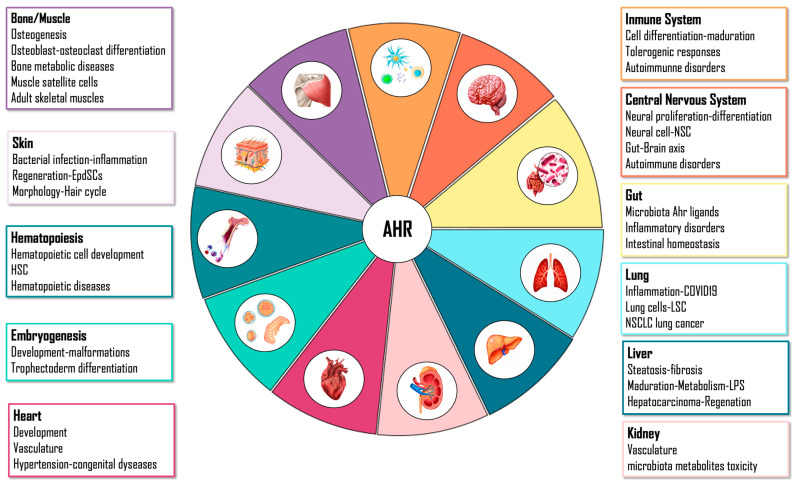
Implication of AHR in organ homeostasis regulation.

Its physiological role may also be justified due to the evidence that different molecules generated during cellular homeostasis can activate its function [105]. This molecular sensor can integrate a wide variety of endogenous and exogenous signals, and the effects of its activation are specific according to binding ligand, cell type, tissue or organ. For this reason, the role of AHR may be so divergent and widespread depending on its physiological context (Figure 2).

### 4.1. Embryo

It has been described how AHR shows different levels of expression in mammalian tissues, with an ubiquitous presence in most of these organisms [106], even from the embryonic development [107]. In fact, AHR has a main role in early embryogenesis, as AHR-null mice embryos show delayed expression of trophectoderm differentiation markers [108], cardiac hypertrophy markers with cardiac enlargement [109], and a zebrafish line with an AHR2 mutation which presents jaw, gill and fin malformations in adult fish [110]. Furthermore, its gestational overactivation leads to altered developmental trajectories, triggering pathological processes in multiple organs and tissues such as lung in rats [111], cleft palate in mice [112], hydronephrosis in mice kidneys [113], prostate and urinary tract dysfunction in mice [114,115,116] and heart and craniofacial malformations in zebrafish [117,118].

### 4.2. Liver

One of the best-characterized phenotypes that provides the most support for the role of AHR in cellular homeostasis is the appearance of marked liver pathology after gene inactivation of the receptor in mice. Thereby, different liver characteristics have been described, including reduced size [119], collagen accumulation resulting in hepatic fibrosis [120], exacerbated proliferation of blood vessels [103] and lack of resolution of the portosystemic connection in adult mice [121]. Recently, the involvement of AHR in the control of hepatic energy and lipid metabolism and its relationship with metabolites from gut microbiome has been discovered [122,123,124,125]. Furthermore, AHR also regulates PI3K, ERK, and Wnt/β-Catenin signaling pathways, which are responsible for proliferation, differentiation and metabolism in the hepatic polyploidization process during liver maturation [61]. This fact is supported by previous studies that show that AHR prevents mitotic progression and induces differentiation, as well as preventing pluripotency in different cell types [28,30,126,127,128]. Moreover, several groups of genes involved in cell differentiation and development are regulated directly by AHR in the absence of an exogenous ligand [129,130].

Additionally, AHR is strongly related to liver stem/progenitor cells [131], with AHR-/- mice showing a more undifferentiated and pluripotent liver phenotype. This absence of AHR increases the susceptibility to hepatocarcinoma development after diethylnitrosamine (DEN) treatment, but also leading to a higher regenerative capacity [132,133]. Therefore, AHR has a direct implication on both processes, since its absence exacerbates cellular senescence markers and increases liver progenitor cells in age-induced hepatocarcinoma [99]. Furthermore, its exogenous modulation by TCDD inhibits hepatic regeneration in mice after partial hepatectomy of the liver [134,135], while hexachlorobenzene (HCB) induces its expression in preneoplastic foci both on rat liver and HepG2 human hepatocarcinoma cell line [136].

### 4.3. Gut

AHR integrates environmental, dietary, microbial and metabolic signals in a ligand- and cell-specific manner. Interestingly, AHR has been found to be regulated by some metabolites from microbiome activity such as short-chain fatty acids and ketone bodies, which can influence host metabolic pathways [137]. In fact, the altered and reduced production of AHR ligands by the commensal flora due to the smoking, antibiotics, oral contraceptives and nonsteroidal anti-inflammatory drugs, have been described to influence inflammatory bowel diseases (IBD) [138,139,140]. Consequently, AHR has started to be considered as a potential target in treatments for the control of intestinal inflammation [141,142,143]. However, its activation has been also reported to lead to proinflammatory effects in the gut [144].

Moreover, AHR signaling plays a main role in the regulation of the proliferation of intestinal stem cells controlling Wnt/β-catenin, EGFR-MAPK/ERK and Notch pathways [145]. However, AHR expression appears to be deregulated in patients with colon cancer according to the cancer genome atlas (TCGA) database (https://xenabrowser.net/, accessed on 25 January 2021), although its endogenous activation by ligands from the diet such as Indole-3-acetic acid (IAA) or β-naphthoflavone (βNF) prevents colon tumorigenesis [146]. The AHR modulation by other ligands has paramount effect in gut because its exogenous activation by 6-Formylindolo [3,2-b]carbazole (FICZ) favors the differentiation of goblet cells enhancing the production of the mucus [144]. Moreover, tryptophan catabolites are responsible for the high production of interleukin 22, providing protection against pathogens [147,148], and in tolerogenic responses in the gastrointestinal tract both in mice and in zebrafish [149]. For these reasons, a correct balance of AHR signaling in this system is needed to maintain a proper physiological homeostasis.

### 4.4. Immune System

The products of tryptophan metabolism derived from bacterial activity mentioned in the previous section affect the activity of different types of immune cells through the activation of AHR [150]. A large body of evidence support a prominent role for AHR in the tolerogenic phenotype and progression of inflammatory and autoimmune diseases. There is an increase in tolerogenic regulatory T cells dependent on the degradation of tryptophan by the enzyme Indoleamine-pyrrole 2,3-dioxygenase (IDO1) and retinoid acid [151,152,153,154] and *Lactobacillus reuteri* (tryptophanase bacterium) stimulates AHR activity and suppresses proinflammatory activities [155,156]. In addition, certain CD4+CD25+Foxp3+ Treg subsets express higher levels of both AHR and Cyp1a1 [157,158]. In fact, AHR-/- models or its inhibition have shown less Treg differentiation [157,159,160,161]. Besides, AHR is connected and works together with CD39, a tolerogenic protein expressed in Treg cells, in the production of IL10 and differentiation of Tr1 cells [162,163,164]. AHR could modulate the gene expression of proinflammatory cytokines, since AHR binding sites have been identified in the promoters of these genes [165,166,167,168], and its activation by TCDD can reverse the demethylation of IL-17 promoters [160]. Moreover, various models of inflammatory and autoimmune diseases have revealed that activation of AHR in vivo by TCDD suppresses allergic responses and immune lung diseases as well as multiple sclerosis and diabetes [150].

### 4.5. Central Nervous System

AHR ligands impact neuronal proliferation, differentiation, and survival and consequently learning skills and memory [169]. It has been found that AHR modifies its gene expression temporally and spatially in the CNS of mice [170]. In addition, AHR is expressed by astrocytes, endothelial cells from the blood–brain barrier and glial cells, regulating CNS integrity and survival [171,172,173]. However, it should be noted that the activation of AHR by various ligands such as TCDD or 3,3′-Diindolylmethane (DIM) can cause neurodegeneration by triggering apoptotic events [174,175,176].

The importance of the “gut-brain axis” has begun to emerge and AHR plays a key role in this connection because microbial ligands have been detected in the brain in physiological conditions [177] and AHR activation by them controls CNS cell function [178,179]. In the experimental autoimmune encephalomyelitis (EAE) model, ITE (2-(1′H-indole-3′-carbonyl)-thiazole-4-carboxylic acid methyl ester) [180,181], I3S (indoxyl-3-sulphate) [182] and TA (tryptamine) [183] induce tolerogenic responses with attenuating neuroinflammation. The reduced level of these agonists is related to neural pathological processes as multiple sclerosis (MS) [182,184,185]. Additionally, the anti-inflammatory effect of AHR ligand Laquinimod, has emerged as a potential clinical treatment for both MS and Huntington disease [186,187].

### 4.6. Skin

The dioxin receptor modulates cell plasticity and migration, and its stimulation by xenobiotics results in severe lesions in the skin as contact hypersensitivity, dermatitis and chloracne. TCDD, the most potent AHR ligand known in humans with a wide range of pathogenic effects, is produced by ultraviolet radiation in the skin [188]. However, it has been described how ligand FICZ improves the skin barrier functions by reducing the local inflammation through involvement of OVO-like proteins [189]. In that context, AHR activation by TCDD (2,3,7,8-tetrachlorodibenzo-p-dioxin) causes chloracne by a mechanism involving stem cells and keratinocytes, modifying gene expression of several genes encoding growth- and differentiation-regulating proteins and its chronic activation produces epidermal hyperplasia and sustained inflammation [190,191]. Furthermore, retinoic acid co-treatment with TCDD aggravates severity of skin lesions in hairless mice via induction of inflammatory response [192]. In line with this, Tapinarof, an AHR agonist derived from bacteria, has recently been reported to have beneficial clinical effects for the treatment of skin inflammation [193]. In contrast, pharmacological inhibition of AHR in human skin equivalents impaired terminal differentiation, epidermal stratification and stratum corneum formation [194]. AHR-/- mice have a patent altered skin homeostasis, regeneration, and hair cycling due to defects in the number and/or functionality of epidermal stem cells (EpdSCs) [195]. Additionally, AHR deficiency in the skin favors bacterial infection with difficulties in controlling stable skin microflora [196,197], while this lack of AHR also protects against UVB-induced cutaneous squamous cell carcinomas [198] or skin tumorigenesis caused by Benzo[a]pyrene [9].

### 4.7. Lung

AHR is considered as a modulator of lung inflammation, function and homeostasis. Most of lung cells show AHR expression, such us macrophages, club cells, alveolar type II cells and endothelial cells [199,200,201]. In particular, AHR participates in barrier function metabolizing components of pollution and other toxic compounds, which can damage lungs. In this way, deficient AHR murine models has been reported to have increased lung inflammation and damage upon exposure to tobacco smoke, lipopolysaccharide and hyperoxia [202,203,204]. Conversely, AHR activation by TCDD has been shown to control secretion of cytokines and infiltration of neutrophils and macrophages which can attenuate inflammation in rodent model of asthma [205] or model of acute hyperoxic lung injury [206]. However, receptor activation by air pollutants and cigarette smoke induce inflammation, contributing to the pathogenesis of chronic obstructive pulmonary disease [207,208,209]. However, lung regeneration has been improved in AHR-/- mice through the expansion of undifferentiated Clara and basal cells and of epithelial cells expressing pluripotency markers Nanog and Oct4 [210].

Regarding non-small-cell lung carcinoma (NSCLC), AHR is considered both a promoter and inhibitor. AHR promotes tumor development by sustaining cell stemness through Jak/Stat3 [211], detoxifying effects of benzo[a]pyrene [212] and regulating CYP1B1 enzyme [213,214]. In the opposite way, AHR represses lung tumors by reducing epithelial-to-mesenchymal transition and invasiveness [215], activation of TGFβ-Smad2 pathway, suppressing lung metastasis [216] and attenuating the oncogenic potential of K-RasG12D [108].

In fact, the involvement of the receptor in the SARS-CoV-2 infection triggered the AHR signaling in lung epithelial cells, leading to the overexpression of mucins and limiting host anti-viral responses mediated by IFN-I and NF-κβ, promoting viral replication [217,218]. Moreover, gene expression data available in gene expression omnibus (GEO) public repository have revealed the activation of AHR signaling following infection by M-CoV, SARS-CoV-1, HCoV-229E, MERS-CoV and SARS-CoV-2 viruses [217].

## 5. Final Thoughts

Since its discovery, AHR has been earning new functions and interactions over the years. As we have addressed in this review, its participation at molecular level involves changes and modifications in the genome structure, epigenetic patterns, regulation of gene expression and signaling pathways, all of them leading to an irrevocable control of tissue/organ physiology and homeostasis. This widespread participation has placed AHR at the core of cell migration, proliferation, pluripotency, tumorigenesis and regenerative processes. Such tendency raises AHR to an almost never-ending source of research in the upcoming years at molecular biology and biomedicine fields.

## Figures and Tables

**Table 1:** List of other AHR-interacting proteins.

Factor	Functional Consequence	Reference
SRC-1, NCoA2, p300/CBP, p/CIP	Coactivators with HAT activity that interact with AHR and/or ARNT to facilitate transcriptional activation	[91]
SHP	Inhibits transcriptional activity of the AHR/ARNT complex	[92]
Brg-1	Histone-modifying factor dependent on ATPase activity and activator of transcription mediated by AHR/ARNT	[93]
Med220, CDK8	Subunits of the mediator complex involved in AHR/ARNT transcriptional activation	[94]
ERα	Functional interactor with AHR in gene regulation	[95]
RB	Direct interaction between Rb and AHR is required for maximal induction of Cyp1a1, suggesting a role of coactivator for RB	[96]
Mybbp1a	Associates with AHR and favors transactivation	[97]
Nedd8	Interacts with AHR increasing its nuclear accumulation and transcriptional activity	[98]
p16, p21	AHR transcriptionally regulates the expression of senescence-related genes	[99]
VEGF, HGF	AHR modulation of angiogenesis through a mechanism that requires VEGF activation in the endothelium	[100]
FGF	Increased fibroblast growth factor (FGF) levels with AHR overexpression.	[99]
Per2 y Bmal1	Circadian rhythms-mediated interaction with AHR. Exposure to TCDD alters their expression pattern.	[101]
Cav-1	AHR modulation of Caveolin-1 in cell migration	[102]
